# Synthetic Cathinone and Cannabinoid Designer Drugs Pose a Major Risk for Public Health

**DOI:** 10.3389/fpsyt.2017.00156

**Published:** 2017-08-23

**Authors:** Aviv M. Weinstein, Paola Rosca, Liana Fattore, Edythe D. London

**Affiliations:** ^1^Department of Behavioral Science, Ariel University, Ariel, Israel; ^2^Department for the Treatment of Substance Abuse, Ministry of Health, Jerusalem, Israel; ^3^Institute of Neuroscience-Cagliari, National Research Council of Italy, Cagliari, Italy; ^4^Department of Psychiatry and Biobehavioral Sciences, University of California Los Angeles, Los Angeles, CA, United States; ^5^Department of Molecular and Medical Pharmacology, University of California Los Angeles, Los Angeles, CA, United States; ^6^Brain Research Institute, University of California Los Angeles, Los Angeles, CA, United States

**Keywords:** synthetic drugs, cathinones, cannabis, amphetamine, new psychoactive drugs

## Abstract

As part of an increasing worldwide use of designer drugs, recent use of compounds containing cathinones and synthetic cannabinoids is especially prevalent. Here, we reviewed current literature on the prevalence, epidemiology, bio-behavioral effects, and detection of these compounds. Gender differences and clinical effects will also be examined. Chronic use of synthetic cathinone compounds can have major effects on the central nervous system and can induce acute psychosis, hypomania, paranoid ideation, and delusions, similar to the effects of other better-known amphetamine-type stimulants. Synthetic cannabinoid products have effects that are somewhat similar to those of natural cannabis but more potent and long-lasting than THC. Some of these compounds are potent and dangerous, having been linked to psychosis, mania, and suicidal ideation. Novel compounds are developed rapidly and new screening techniques are needed to detect them as well as a rigorous regulation and legislation reinforcement to prevent their distribution and use. Given the rapid increase in the use of synthetic cathinones and cannabinoid designer drugs, their potential for dependence and abuse, and harmful medical and psychiatric effects, there is a need for research and education in the areas of prevention and treatment.

## Introduction

Over the past decade, there has been a worldwide increase in the opportunity for use and consumption of Novel Psychoactive Substances (NPS) – which produce “legal highs” (1–8). Usually mimicking the psychoactive effects of illicit drugs of abuse, these “designer” drugs vary widely in composition, and are mainly sold online and from street retailers. Some of the drugs are advertised as being legal and are often labeled as “not for human consumption” or “licensed by the Ministry of Health,” to bypass—legislation enforcement. Based on their pharmacological mechanisms and psychoactive properties, designer drugs fall into four major categories: (i) amphetamine-like stimulants, which include cathinones derivatives and piperazine derivatives that are sold as substitutes for “ecstasy,” (ii) synthetic cannabinoids, (iii) hallucinogenic/dissociative agents, and (iv) opioid-like compounds (9). While new compounds are appearing relentlessly on the drug market, information regarding their potential toxicity is scarce and the number of emergencies related to their use is increasing (10, 11). Given the public health threat that is growing in complexity, a multidisciplinary coordinated effort is required to elucidate the acute and chronic effects of synthetic drugs of abuse (12). According to the World Drug Report 2016 (13), the majority of the substances reported for the first time between 2012 and 2014 were synthetic cannabinoids, followed by synthetic cathinones that have been steadily increasing since their first appearance (2010). Synthetic cathinones and synthetic cannabinoids have been studied more extensively than other classes of NPS (e.g., new hallucinogens and opioid-like compounds) over the past 10 years. These two classes of substances are the most popular, both induce central and peripheral serious effects and activate the brain reward system thus showing to possess abuse potential (14, 15). Since they pose a major health hazard potential, this review will focus on these two classes of NPS.

Synthetic cathinones are psychostimulants related to the naturally occurring parent compound cathinone (16, 17), a monoamine alkaloid found in the khat plant (*Catha edulis*), a flowering plant native to the Horn of Africa and the Arabian Peninsula that is being chewed in these areas for thousands of years. Cathinone is a psychoactive substance known to cause excitement, loss of appetite, and euphoria. As members of the phenethylamine class of drugs, they are structurally and pharmacologically similar to amphetamine and 3,4-methylenedioxymethamphetamine (MDMA). The most commonly used drugs in this class have been 4-methylmethcathinone (mephedrone), 3,4-methylenedioxy-*N*-methylcathinone (methylone), and 4-methylenedioxypyrovalerone (MDPV), although MDPV and mephedrone are no longer prevalent (16, 17). Reports of abuse of cathinone derivatives date back to the 1990s, when mephedrone was the first designer drug of this class (18). Mephedrone is the most widely abused synthetic cathinone in Europe, and MDPV and methylone are the most frequently abused synthetic cathinones in the United States (7). These drugs have been commonly referred to as “bath salts,” “plant food,” or “fertilizer,” because they were at times disguised and commonly included in products that were labeled and sold as such (7, 9). New analogs, legal to possess, at least until they are formally banned, are frequently introduced, and it has been estimated that nearly 250 new analogs are produced each year (19). These drugs are consumed by oral ingestion, inhalation, and snorting. Notably, the recent trend to supplement use of more conventional psychostimulants, such as amphetamine and cocaine, with mephedrone, may lead to serious psychotic, neurological, cardiovascular, and sexual health consequences (4).

Synthetic cannabinoid receptor agonists, which often mimic the effects of marijuana, are added to herbs, such as *Damiana* leaves, and sold under different brand names, such as “Spice” or Mr. Nice Guy in Europe since 2006 (20, 21). These products are also called “K2,” “herbal incense,” “Cloud 9,” “Mojo,” and with many other names (22). Advertised as “exotic incense blends which release a rich aroma,” Spice, and Spice-like preparations in Europe have been found to contain different substances with various chemical structures, including those (i) based on over 450 compounds originally produced for medical research by John W. Huffman (e.g., JWH-018), (ii) HU-210 developed by Raphael Mechoulam at the Hebrew University, and (iii) the cyclohexylphenol (“CP”) cannabinoids developed at Pfizer Pharmaceuticals (23). AM-2201, an indole derivative, which differs from JWH-018 by a fluorine atom in the pentyl chain, is commonly found in Korea and has nanomolar affinity for cannabinoid receptors (24). After the appearance on the drug market of over 100 compounds that activate cannabinoid receptors, new compounds with different chemical structures that directly or indirectly stimulate the cannabinoid CB1 receptors are expected. The perceived harmfulness of synthetic cannabinoids among secondary school students (twelfth grade) increased between 2012 (the first year of measurement) and 2014, which may have contributed to the decline in use (9). This review is divided into the following categories: epidemiology of use, pharmacology, neuropsychiatric findings, other medical conditions, and regulation.

## Epidemiology

### Synthetic Cathinones

Masticating khat leaves has been a social habit among Saudi Arabian and East African cultures, for several centuries. Cathinone is the main psychoactive ingredient that was detected in the leaves of the *C. edulis*. In the Middle East and in East Africa khat use is still common, more among men than women, although the gap is narrowing (25). Yet, although both sexes typically report to use khat to upkeep of tradition, men’s use is more frequently associated to recreational purposes, while women often report to use it to treat headache or lose weight (26).

Recent surveys have considered the problem of synthetic cathinones use in the United States. In an online survey of 113 participants, who reported use of synthetic cathinones, respondents were males, with age range of 18–24 years, and Caucasian holding college education (27). Their use in the past year was low (≤10 days), but recurrent. The intranasal route of administration was most frequently reported, and its effects were similar to those of cocaine and amphetamines. Synthetic cathinones increased sexual desire and sexual risk-taking behavior. More than half of the responders met DSM-V diagnostic criteria for a substance-related disorder. Self-reported prevalence of the use of synthetic cathinones was less than that of marijuana, cocaine, *Salvia divinorum*, synthetic cannabinoids, methamphetamine, and MDMA. In another survey, reaching over 2,300 students at a large university in the Southeastern United States, 1.07% of respondents endorsed ever using synthetic cathinones, and those who did were more often men than women, Hispanics, and Native American rather than Caucasian students, and athletes more than non-athletes (27).

Studies of synthetic cathinones use in Europe have also been informative. In Hungary, there has been a shift among street drug users from the use of heroin to mephedrone injection, potentially increasing the risk of severe psychiatric symptom profile and increased possibility of dependence (28). Among 1,000 individuals who completed a survey in schools and universities in Scotland (49.8% males and 50.2% females), 20.3% reported previous use mephedrone, with 23.4% reporting single use and 4.4% daily use (29). In a survey of 249 NPS users interviewed in open public places, clubs, and discotheques in Slovenia, 67.9% of the respondents endorsed having tried either synthetic cathinones or amphetamines (30). Of those who reported using synthetic cathinones or amphetamines, most reported having used 3-methylmethcathinone first, 43.0% had first used methylone, and 37.3% had first used mephedrone. Users have associated the new drugs with high risks and favored traditional drugs. A report on the occurrence and trends of new synthetic drugs in Sweden included participants who were 13–63 (median 20) years of age (31). The report described a widespread use of NPS in adolescents and young male adults (79%), in incidents of drug intoxications reported by emergency departments and intensive care units. Of the 189 blood and urine samples that were examined in the laboratory, 156 (83%) samples were found positive for NPS. More than 50 new synthetic drugs were detected. These included synthetic cannabinoid receptor agonists (“Spice”), piperazines, substituted phenethylamines, synthetic cathinones, hallucinogenic tryptamines, piperidines, opioid-related substances, ketamine and related substances, and γ-aminobutyric acid analogs. About half of the cases involved multiple drug intoxications, making it hard to link the clinical presentations with one specific substance.

### Synthetic Cannabinoids

Synthetic cannabinoid users are usually men in their twenties, who use also other drugs. In a study of adult marijuana and tobacco users, the 42 respondents were male young adults, high school graduates, who also smoked tobacco and cannabis (86% smoked cannabis on five or more days per week) (32). A very high proportion (91%) were familiar with synthetic cannabinoid products, half (50%) smoked them previously, and a minority (24%) used them over the last month. Common reasons for use included expecting a strong “high” while avoiding detection by urine toxicology. The main side effects were difficulties in thinking, anxiety, dry mouth, and headache. Students also used synthetic cannabinoids out of curiosity and for feeling “high” (33). Eleven adolescents experienced euphoria and negative effects on memory, and nine reported negative mood changes (34).

Several informative surveys of synthetic cannabinoids use have involved college students. In a study of 852 college students in the United States, 69 (8%) reported using the drugs at least once, and more common use in males and in the first or second year of college (35). A survey of students from a local health clinic and a US University found 9, 5, and 3% lifetime, past-year, and past 30-day use of synthetic cannabinoids, respectively (36). In Rhode Island, 1,080 young (18–25 years old) participants were surveyed between January 2012 and July 2013, and 9% reported use of synthetic cannabinoids in the last month. Synthetic cannabinoid users were predominantly males who did not attend school, smoked cigarettes, were binge alcohol drinkers, who used marijuana daily, as well as other recreational drugs (37). A survey of over 3,100 college students in North Carolina and Virginia showed that lifetime prevalence of synthetic cannabinoids was 7.6% at college entry and 6.6% first use during college (38). During the fourth year of college, lifetime synthetic cannabinoids use was reported by 17.0%. The “Monitoring the Future study,” a nationally representative sample of high school seniors between 2011 and 2013 included almost 12,000 participants of mean age 18 (39). In this sample, 10% reported any recent use and 3% reported frequent use (over six times) of synthetic cannabinoids. Females were less likely to use synthetic cannabinoids, and going out 4–7 evenings per week increased likelihood of drug use. Several factors were identified as increasing the risk for synthetic cannabinoid use, mainly high frequency of lifetime marijuana use, lifetime use of alcohol, cigarettes, and other illicit drugs. Among 396 patients entering residential treatment for Substance Use Disorder in the United States, 150 reported using synthetic cannabinoids in their lifetime. Motives for drug use were curiosity (91%), “feeling good” or “getting high” (89%), relaxation (71%), and “getting high” while avoiding detection (71%) (40). According to the 2009–2013 US National Survey of Drug Use and Health, the gender gap in the self-reported use of synthetic cannabinoids is smaller compared to other categories of NPS among non-institutionalized individuals aged 12–34 years old (41).

In an anonymous online survey among almost 15,000 participants in the UK, 2,513 (17%) reported use of synthetic cannabinoids. Among them, 41% reported use over the last 12 months (42). Almost all synthetic cannabinoids users (99%) used natural cannabis at least once. Synthetic cannabinoids had a shorter effect and faster time to peak effect than natural cannabis. Most users (93%) preferred natural cannabis to synthetic cannabinoids. Natural cannabis had greater pleasurable effects when “high” and allowed better function after use. Synthetic cannabinoids had negative effects such as hangover, and paranoia. In an anonymous follow-up online survey of drug use with over 22,000 respondents, use of synthetic cannabinoids was estimated as 30 times as more risky than natural cannabis (43).

In Australia, a sample of 316 synthetic cannabinoid users (77% male, mean age 27 years) reported mean synthetic cannabinoid use of 6 months, 35% reported weekly use and 7% reported daily use (44). Reasons for first use included: curiosity (50%), legality (39%), availability (23%), recreational effects (20%), therapeutic effects (9%), avoidance of detection by standard drug screening tests (8%), and aid for the reduction or cessation of cannabis use. In a further study of over 1,100 students (mean age 14.9 years) from secondary schools in Australia, 2.4% had ever used synthetic cannabis and 0.4% had used synthetic stimulants (45). Users also had an episode of binge drinking in the past 6 months, used tobacco and had higher levels of psychological distress and lower perceived self-efficacy to resist peer pressure than non-users, but did not differ from users of other illicit drugs.

### Comparative Epidemiology

In a survey covering the years 2009 to 2012 in the United States, synthetic cathinone and synthetic cannabis exposures totaled 7,467 and 11,561, respectively (46), with increases in the use of both from 2009 to 2011. Synthetic cathinone use increased from none reported in 2009, to 298 in 2010, and 6,062 in 2011. By comparison, there were 14 reported synthetic cannabis exposures in 2009, 2,821 in 2010, and 6,255 in 2011. The number of those who were first-time exposed to synthetic cathinones was lower in 2012 (1,007) than in 2011 (2,027), while the number of first-time exposures to synthetic cannabis was higher in 2012 (2,389) than in 2011 (1,888) possibly reflecting a shift from synthetic cathinone use toward the use of synthetic cannabinoids. Most exposures occurred in the Midwest and Southeastern US (64.8% of synthetic cathinone and 58% of synthetic cannabis exposures). Males comprised 69% (*n* = 5,153) of synthetic cathinone users and 74% (*n* = 8,505) of synthetic cannabis users. Use of synthetic cathinones was highest in individuals 20–29 years of age (*n* = 2,943), while use of synthetic cannabinoids was highest for younger respondents, i.e., individuals 13–19 years of age (*n* = 5,349). A recent study involving 1,740 young adults recruited in US nightlife scenes (18–40 years old, mean age 26.4) showed that use of mephedrone and synthetic cannabinoids adults in US nightlife scenes is lower than in EU nightlife scenes (47). Specifically, 8.2% used synthetic cannabinoids, 1.1% used mephedrone. Gay and bisexual men reported more frequent use of mephedrone and more frequent use of synthetic cannabinoid use in individuals with Latin origin. In conclusion, synthetic cannabis emerged first with overall more reported exposures than synthetic cathinone. In 2012, synthetic cathinone abuse declined while synthetic cannabis abuse increased. Young men intentionally abusing synthetic cannabinoids *via* inhalation make up the majority of users.

## Drug Pharmacology, Central Effects, and Clinical Features

### Synthetic Cathinones

Synthetic cathinones represent a broad class of pharmacologically active compounds that induce numerous effects with different mechanisms of action. As such, each case of synthetic cathinone intoxication should be evaluated separately, at least until preclinical research will provide structure–activity relationships for each compound. Similar to the action of other psychostimulants, synthetic cathinones have an effect on plasma membrane transporters of the monoamine neurotransmitters, dopamine, norepinephrine, and serotonin (48). Mephedrone and methylone, but not MDPV, act as non-selective transporter agonists, thereby promoting release of all of these neurotransmitters. Conversely, MDPV acts as a potent blocker at catecholamine transporters with little effect at the serotonin transporter (48). Mephedrone or methylone administered to rats increase extracellular concentrations of dopamine and serotonin in the brain, similar to the effects of MDMA (48). Synthetic cathinones elicit locomotor stimulation in rodents similar to other psych-stimulants. The enhanced dopamine transmission by synthetic cathinones presents a high potential for addiction that may result in adverse effects (49). See Table 1 for behavioral and pharmacological effects of synthetic cathinones in rodents.

**Table 1:** Studies investigating the behavioral effects of synthetic cathinones.

Animals	Synthetic cathinone tested	Main findings	Reference
Male ICR mice	α-PBP, α-PPP, α-PVP	Motor stimulation	(50–52)
	MDPV	Decreased motor coordination	
	MEPH	Ataxia	
	Methylone		
	3-FMC, 4-FMC, 4-MePPP		

Male Sprague-Dawley rats	α-PVP	Significantly lower ICSS threshold	(53–55)
	MDPV		
	MEPH		
	Methcathinone		
	Methylone		
	R-MEPH, 4-MEC		

Male rats (Wistar, Sprague-Dawley)	α-PVT	Sustain IVSA behavior	(56–63)
	BMAPN		
	Buphedrone		
	MACHP		
	Methylone		
	MPDV		
	4-MEC, 4-MePPP		

Male mice (CD-1, ICR, C57BL/6J or Swiss Webster)	α-PBP, α-PVP, α-PVT	Induce CPP	(58–62, 64, 65)
	BMAPN		
	Buphedrone		
	MACHP, MDPV		
	MEPH, MAOP		
	Methylone		
	PIPP		

Male and female (MDPV) Sprague-Dawley rats	MDPV	Induce CPP	(55, 66–68)
	R-MEPH		
	4-MEC		

Male Sprague-Dawley rats	α-PBP, α-PVP, α-PVT	Substitute for the discriminative stimulus effects of METH in a DD paradigm	(61, 65, 69, 70)
	Methcathinone		
	Pentedrone		
	Pentylone		
	3-FMC, 4-MePPP, 4-MEC		

*α-PBP, α-pyrrolidinopropiobutiophenone; α-PVP, alpha-pyrrolidinovalerophenone α-PVT, alpha-pyrrolidinopentiothiophenone; buphedrone, [2-(methylamino)-1-phenylbutan-1-one, α-methylamino-butyrophenone]; BMAPN, 2-(methylamino)-1-(naphthalen-2-yl) propan-1-one; CPP, conditioned place preference; DD, drug discrimination; ICSS, intracranial self-stimulation; IVSA, intravenous self-administration; MACHP, [1] 2-cyclohexyl-2-(methylamino)-1-phenylethanone; MAOP, [2] 2-(methylamino)-1-phenyloctan-1-one; MEPH, mephedrone; METH, metamphetamine; MDPV, methylenedioxyphyrovalerone; PIPP, f α-piperidinopropiophenone; R-MEPH, R-mephedrone; 3-FMC, 3-fluoromethcathinone; 4-FMC, 4-fluoromethcathinone; 4-MEC, 4-methylethcathinone; 4-MePPP, 4-methyl-alpha-pyrrolidinopropiophenone*.

#### Acute and Chronic Side Effects

Acute administration of low doses of synthetic cathinones produces euphoria and increases alertness, but high doses or chronic use can result in serious adverse effects, such as hallucinations, delirium, hyperthermia, and tachycardia (70). Repeated use of synthetic cathinones is associated with paranoia and hallucinations and some patients developed “excited delirium,” a syndrome with symptoms of extreme agitation and violent behavior (70). The symptoms included dehydration, muscle damage and renal failure that may result in multi-organ failure and death. A 40-year-old male, who had no previous psychiatric history developed “excited delirium” after ingesting “bath salts” with psychosis and violence (71). Forty-three postmortem cases with detected synthetic cathinones had the following associated causes of death: driving under the influence of drugs (17 cases), domestic violence (2 cases), suicide (4 cases), overdose (12 cases), accidents (6 cases), drug-facilitated assault (1 case), and homicide (1 case) (72). The highest measured MDPV and methylone concentration was detected in a case of suicide by hanging and in a driver, respectively. A single case of death following methylone ingestion was reported in France (73).

Cardiovascular effects (tachycardia, hypertension) and hallucinations are the most recurrent medical complications of synthetic cathinone use. The most frequently reported unwanted clinical effects among cases reported to Texas poison centers during 2010–2011 were tachycardia (45.9%), agitation (39.2%), hypertension (21.0%), hallucinations (17.7%), and confusion (13.0%) (74). A study of “bath-salt” exposure conducted in two poison centers in the United States found primarily neurological and cardiovascular effects. Drugs effects included agitation, combative behavior, tachycardia, hallucinations, paranoia, confusion, chest pain, myoclonus, hypertension, mydriasis, elevations in creatine phosphokinase, hypokalemia, blurred vision, and death (8). Signs of severe toxicity, such as hyperthermia, metabolic acidosis and prolonged rhabdomyolysis, indicative of high serotonergic activity, were also reported (75). A single case described cardiovascular manifestations, including tachycardia, hypertension, myocardial infarction, arrhythmias, and cardiac arrest (76).

#### Seizures and Withdrawal

Synthetic cathinone exposure has also resulted in many cases of seizures in the pediatric population. The American Association of Poison Control Centers database was used to examine synthetic cathinone exposures in children and youth below 20 years of age between 2010 and 2013 (77). There were 1,328 cases of pediatric synthetic cathinone exposures, of which 73 cases presented seizures complications: 37 (50.7%) involved a single seizure, 29 (39.7%) involved multiple seizures, and seven (9.6%) developed epilepsy. Fever and acidosis were associated with single seizures, multiple seizures, and status epilepsy. There were no correlations between seizure activity and electrolyte abnormalities, hallucinations and/or delusions, tachycardia, or hypertension. The most commonly co-ingested substances were tetrahydrocannabinol, alcohol, and opioids. Finally, in Italy, a baby born to a woman who was a chronic consumer of 4-methylethcathinone showed symptoms of neonatal withdrawal syndrome (78). The newborn presented with increased jitteriness and irritability, high-pitched crying, limbs hypertonia and brisk tendon reflexes (78).

Regrettably, there is little information regarding the pharmacokinetics of synthetic cathinones in preclinical or clinical studies. Studies conducted so far have demonstrated that, upon systemic administration, synthetic cathinones are metabolized in several phase I compounds, some of which have been found to be substrates at monoamine transporters when assessed *in vitro* or to exert neurochemical actions *in vivo* (79, 80). The resulting metabolites can also partially undergo phase II metabolism (81). The presence of active metabolites could account, at least in part, for the diversified effects of these drugs.

### Synthetic Cannabinoids

Unlike Δ9-tetrahydrocannabinol (THC), synthetic cannabinoids are extremely potent, highly efficacious, full agonists of the cannabinoid receptors (82, 83), including CB1 receptors in the brain (84–89). There is substantial variability in the molecular constituents of different compounds, between assortment of the same product, and even within a package (20). In addition to synthetic cannabinoids, Spice drugs may contain preservatives, additives, fatty acids, amides, esters, the benzodiazepine phenazepam and *O*-desmethyltramadol, an active metabolite of the opioid medication tramadol (90–92). Studies in rodents have indicated that most synthetic cannabinoids produce effects and toxicity that, overall, are similar to those of THC and include hypothermia, analgesia, hypo-locomotion, and akinesia (93). Yet, most effects are more potent and long-lasting than THC. See Table 2 for motor and reward-related behavioral effects of synthetic cannabinoids in rodents.

**Table 2:** Studies investigating the behavioral effects of synthetic cannabinoids.

Animals	Synthetic cannabinoid tested	Main findings	Reference
Male mice (C57BL/6J, Swiss Webster)	“Buzz” (5.4% JWH-018)	Induces a dose-related tetrad effects similar to marijuana/THC	(94, 95)
	JWH-018		
	JWH-073		

Male Swiss Webster/ICR mice	AB-FUBINACA	Decrease locomotor activity	(96–99)
	AM-2201	Induce catalepsy	
	APINACA/AKB-48		
	JWH-018, JWH-073, JWH-200, JWH-203 JWH-250		
	PB-22 (QUPIC)		
	UR-144, XLR-11, 5F-PB-22		

Male ICR mice	JWH-018	Significantly impairs sensorimotor functions	(14, 99)
		Induced convulsions, myoclonia and hyperreflexia (at high doses)	

Male Sprague-Dawley rats/C57BL/6 mice	JWH-018	Sustains IVSA behavior	(100)

Male mice (ICR)	JWH-073, JWH-081, JWH-210	Induce CPP	(101)

Male Sprague-Dawley rats	JWH-175	Induces CPP	(102)

Male ND4 Swiss–Webster/ICR mice	AB-CHMINACA	Fully substitute for THC in a DD paradigm	(98, 103)
	AB-PINACA		
	ADBICA		
	ADB-PINACA		
	FUBIMINA		
	JWH-018, JWH-122, JWH-210		
	RCS-4, THJ-2201		

Male and female (JWH-018) Sprague-Dawley rats	AB-FUBINACA	Fully substitute for THC in a DD paradigm	(96, 97, 104)
	AM-2201		
	APINACA/AKB-48		
	JWH-018, JWH-073, JWH-200, JWH-203, JWH-250		
	PB-22/QUPIC		
	UR-144, XLR-11, 5F-PB-22		

Male adolescent rhesus monkeys	JWH-018	Shows discriminative stimulus effects; dose-dependently increases drug-lever responding and decreased response rate	(105)

Female and male adult rhesus monkeys	AM-2201	Substitute for the discriminative stimulus effects of Δ9-THC	(106)
	JWH		
	JWH		

*AB-CHMINACA, N-[1-amino-3-methyl-oxobutan-2-yl]-1-[cyclohexylmethyl]-1H-indazole-3-carboxamide; AB-FUBINACA, N-(1-amino-3-methyl-1-oxobutan-2-yl)-1-(4-fluorobenzyl)-1H-indazole-3-carboxamide; AB-PINACA, N-(1-amino-3-methyl-1-oxobutan-2-yl)-1-pentyl-1H-indazole-3-carboxamide; ADBICA, N-(1-amino-3,3-dimethyl-1-oxobutan-2-yl)-1-pentyl-1H-indole-3-carboxamide; ADB-PINACA, N-[1-(aminocarbonyl)-2,2-dimethylpropyl]-1-pentyl-1H-indazole-3-carboxamide; AM-2201, [1-(5-fluoropentyl)-1H-indol-3-yl]-1-naphthalen-methanone; APINACA/AKB-48, N-(1-adamantyl)-1-pentyl-1H-indazole-3-carboxamide; CPP, conditioned place preference; DD, drug discrimination; FUBIMINA, (1-(5-fluoropentyl)-1H-benzo[d]imadazol-2-yl)(naphthalen-1-yl)methanone; ICSS, intracranial self-stimulation; IVSA, intravenous self-administration; JWH-018,1-pentyl-3-(1-naphthoyl)indole; JWH-073, naphthalen-1-yl-(1-butylindol-3-yl)methanone; JWH-081, 4-methoxynaphthalen-1-yl-(1-pentylindol-3-yl)methanone; JWH-175, (1-pentylindol-3-yl) naphthalen-1-ylmethane; JWH-200, [1-[2-(morpholinyl)ethyl]-1H-indol-3-yl]-1-naphthalenyl-methanone; JWH-203, (2-(2-chlorophenyl)-1-(1-phentyl)-1H-indol-3-yl)-methanone; JWH-210, 4-ethylnaphthalen-1-yl-(1-pentylindol-3-yl)methanone; JWH-250, 2-(2-methoxyphenyl)-1-(1-pentylindol-3-yl)methanone; PB-22/QUPIC, quinolin-8-yl 1-pentyl-1H-indole-3-carboxylate; RCS-4, 2-(4-methoxyphenyl)-1-(1-pentyl-indol-3-yl)methanone; THJ-2201, [1-(5-Fluoropentyl)-1H-indazol-3-yl](naphthalen-1-yl)methanone; UR-144, (1-pentylindol-3-yl)(2,2,3,3-tetramethylcyclopropyl)methanone; XLR-11, 5F-UR-144[1-(5-fluoro-pentyl)-1H-indol-3-yl](2,2,3,3-tetramethylcyclopropyl)methanone; 5F-PB-22, quinolin-8-yl-1-(5-fluoropentyl)-1H-indole-3-carboxylate*.

#### Psychosis

Cannabis use has potential for inducing psychosis [for recent reviews, see Ref. (107, 108)], and it would be reasonable to expect synthetic cannabinoids to have the same effect. Because of their high potency and the fact that synthetic cannabinoids act as full cannabinoid receptor agonists, even short or occasional use of these synthetic compounds can produce unwanted effects, such as insomnia, memory impairment, headaches, dizziness, and delusions. Moreover, unlike natural cannabis, synthetic cannabinoids contain no cannabidiol that may be protective against psychosis. Cannabidiol antagonizes the psychotomimetic and other psychotropic effects of THC although the mechanisms underlying its therapeutic effect are still not clear (109). Compared with natural cannabis, synthetic cannabinoids may cause more frequent and more severe unwanted negative effects, and may have high-risk for psychosis especially in young users (110). Case reports have documented psychosis (111, 112), mania (113), and suicidal ideations (114) in synthetic cannabinoid users.

#### Brain Imaging Studies

Although brain imaging studies have pointed to abnormalities in cerebral perfusion, deficits in brain volume and white-matter pathways (115), brain imaging has scarcely been applied to understand the neural correlates of synthetic cannabis use. A comparison of 20 male patients who had used synthetic cannabinoids with 20 healthy male controls indicated that drug users had smaller gray-matter volume in the thalamus and the cerebellum (116). A single case study of a 23-year-old patient reported severe withdrawal syndrome upon voluntary abstinence from “Spice Gold.” Craving, affective symptoms and a range of somatic complaints were reported, but these were resolved after several days of monitored abstinence (117). In this patient, dopamine D_2_ and D_3_ receptor availability was 20% lower in the striatum and in extra-striatal regions with respect to healthy control participants, but returned to control values with detoxification. Brain imaging studies suggest that synthetic cannabinoid use can produce remarkable changes in the brain, but they are still preliminary, and the extent and duration of the neural sequelae of synthetic cannabinoid use remains to be determined.

#### Effects on Driving

Reports concerning driving under the influence of synthetic cannabinoids also reflect their impact on the nervous system. One report from the United States indicated that drivers under the influence of synthetic cannabinoids had slow and slurred speech, and poor coordination (118). A survey in Germany found behavioral deficits that were moderate except for worsening of paranoia in one case (119). The symptoms were similar to the effects of cannabinoid agonists but could also be a result of alcohol or other drugs detected by blood analysis. In several case reports sedating effects and impairment of fine motor skills were noted (120). In Poland, a single case showed that use of a synthetic-containing product caused effects and impairment similar to THC (121). Very few cases of synthetic cannabinoids were detected in the blood of drivers in Norway (122, 123).

#### Health Hazards and Withdrawal

Synthetic cannabinoid use has been associated with serious hazardous health effects on multiple systems, and with death (124, 125). For example, among 3,572 calls related to synthetic cannabinoid use to call centers in the United States, 2,961 had a medical outcome, 11.3% callers had a major adverse effect, and 15 deaths were reported (126). The most common side effects are tachycardia, agitation, irritability, confusion, dizziness, drowsiness, hallucinations, delusions, hypertension, nausea, vomiting, vertigo and chest pain (127). Central nervous system effects range from headache to coma and included seizures, myoclonus, catatonic stupor, cerebral ischemia, and encephalopathy (128–131). Case reports have documented cardiac complications, ranging from chest pain (132) to myocardial infarction (133, 134), and cardiac arrest (135–137). Cases of acute kidney damage and renal failure following use of synthetic cannabinoids have also been reported (138). Dyspnea, rhabdomyolysis, diaphoresis, and hypokalemia, which are not commonly reported by cannabis users, have been associated with synthetic cannabinoid use (22). Case reports have also described respiratory depression following synthetic cannabinoids use (139) and, with chronic use, also pulmonary complications and pneumonia (140, 141). Rare cases of cannabinoid-induced hyperemesis syndrome were described which included repeated nausea and vomiting, abdominal pain, and a compulsion to take hot showers (142, 143).

Prolonged habitual use of synthetic cannabinoids resulted in withdrawal syndrome in case reports and in a study of 47 patients admitted to detoxification services (144, 145). The symptoms were similar to those of withdrawal from THC, including anxiety, myalgia, chills, anorexia, mood swings and tachycardia, but were more severe and did not seem to improve with the administration of THC (144, 146). The differences in presentation may reflect the inclusion of extraneous compounds, including amphetamine-like stimulants.

Noteworthy, differently from marijuana, while THC is metabolized to one active metabolite only, metabolism of new synthetic cannabinoids leads to the generation of pharmacologically active metabolites that remain biologically active and hold high affinity for the cannabinoid CB1 receptor (147, 148). As for synthetic cathinones, these active metabolites may prolong the psychotropic effects of the parent compound thus contributing to its toxicity.

## Regulation and Legislation

Responding to the rapid appearance of NPS with molecular structures that have not been covered by legislation, the governments of several countries have recognized the need for new mechanisms of control with accelerated ways to curtail the free sale and distribution of these substances (149). In Europe, since 1997 three levels of control were introduced: Early Warning System, risk assessments of newly emerged substances performed by the European Monitoring Centre for Drugs and Drug Addiction (EMCDDA) scientific committee and European Council decisions advocating new legislations (132). The possession, use, and synthesis of synthetic cathinones became subject to legal classification in Europe in 2010 (150) and in the United States in 2011 (149). Some countries such as Denmark, the UK, and Israel (151) opted for “temporary bans” of new psychoactive substances considered to pose danger to public health during which a risk assessment of a particular compound could be performed thus facilitating its subsequent inclusion into the Dangerous Drugs Ordinance. Other countries such as New Zealand, Ireland, Poland, and Romania (152) chose “pre-market approval” regulation regime for synthetic drugs that pose low health risk on the base of preclinical and clinical evidence. The effectiveness of these legal measures and regulations on the selling and marketing of the new psychoactive substances has still to be assessed.

## Conclusion

Synthetic cathinones and synthetic cannabinoids became increasingly popular despite the potential harms associated with their use. Synthetic cathinones have similar clinical effects to amphetamines and MDMA whereas synthetic cannabinoids are high-potency, full agonists at cannabinoid receptors and induce THC-like effects, but more potent and enduring. Both classes of substances have various adverse health effects. Synthetic cathinones cause anxiety, agitation, panic, dysphoria, psychosis, and bizarre behavior whereas synthetic cannabinoids cause agitation, irritability, confusion, hallucinations, delusions, psychosis, and death (as well as other health problems illustrated above in the text and in Tables 1 and 2).

Chronic use of synthetic cathinones and synthetic cannabinoids results in adverse medical and psychiatric effects that seem to be higher than those induced by the natural parent compounds (i.e., cathinone and THC). In comparison with other known amphetamines, synthetic cathinones such as MEPH and MDPV exhibit a pharmacological profile that is more typical of methamphetamine and cocaine, respectively, while methylone shows a pharmacological profile that more closely resembles MDMA; yet, clinical toxicology of synthetic cathinones is not yet fully characterized. Synthetic cannabinoids show higher toxicity compared with natural cannabinoids, and their long-term effects are still to be investigated. In view of the increasing demand for these substances and their severe associated risks, more rigorous research on the effects of synthetic cathinones and synthetic cannabinoids is urgently required in order to understand their pharmacological effects and assist clinicians in managing adverse events.

These two classes of synthetic drugs are composed of pharmacologically diversified compounds with multiple mechanisms of action. Preclinical studies are urgently needed to elucidate their single action in both the brain and the periphery as well as their synergistic effects and long-term consequences. At clinical level, development of combined and integrated pharmacological and psychological approaches to treat intoxication symptoms is necessary. Treatment protocols currently available for the better-known parental drugs need to be adapted to face with the increasing number of intoxications reported after the use of synthetic cathinones and synthetic cannabinoids.

## Author Contributions

AW and LF contributed substantially to the conception and design of the review. All the authors contributed to further drafts of the manuscript, critically revised, and approved the final version of the manuscript.

## Conflict of Interest Statement

The authors declare that the research was conducted in the absence of any commercial or financial relationships that could be construed as a potential conflict of interest.
